# HOXC-AS1-MYC regulatory loop contributes to the growth and metastasis in gastric cancer

**DOI:** 10.1186/s13046-019-1482-7

**Published:** 2019-12-23

**Authors:** Yangyang Dong, Xinyu Li, Zhibin Lin, Wenbing Zou, Yan Liu, Huiyang Qian, Jing Jia

**Affiliations:** 2nd Department of Gastrointestinal Surgery, Quanzhou First Hospital Affiliated to Fujian Medical University, 248-252 East Street, Licheng District, Quanzhou City, 362000 Fujian Province China

**Keywords:** HOXC-AS1, Gastric cancer, MYC, miR-590-3p, BRG1

## Abstract

**Background:**

Gastric cancer (GC) is one of the most prevalent and deadly malignancies worldwide. Accumulating reports have indicated the participation of long non-coding RNAs (lncRNAs) in the onset and progression of GC.

**Methods:**

GSE109476 data was utilized to screen out lncRNAs dysregulated in GC. Gene expressions were determined by qRT-PCR and western blot. Both in vitro and in vivo experiments were carried out to assess the function of HOXC-AS1 in GC. The association between genes was verified via RIP, ChIP, CoIP, RNA pull down and luciferase reporter assays, as appropriate.

**Results:**

HOXC-AS1 was discovered to be upregulated in GC and located both in cytoplasm and in nucleus in GC cells. Functionally, inhibition of HOXC-AS1 restrained GC cell growth and metastasis both in vitro and in vivo. Moreover, HOXC-AS1 was proved to be trans-activated by c-MYC in GC. In return, HOXC-AS1 positively regulated MYC expression in GC through targeting miR-590-3p/MYC axis in cytoplasm and modulating BRG1/β-catenin complex-activated MYC transcription in nucleus. Furthermore, the rescue assays verified that MYC mediated HOXC-AS1-affected GC progression.

**Conclusion:**

Our research illustrated a feedback loop of HOXC-AS1-MYC in aggravating GC cell growth and metastasis, highlighting HOXC-AS1 as a promising target for GC diagnosis and treatment.

## Introduction

Gastric cancer (GC) is the most prevalent and deadly cancer type in gastrointestinal system around the world [1, 2]. To date, surgical resection is undisputedly the only strategy to cure GC patients, while most of the cases have developed into advanced stages and are unsuitable for surgery [3, 4]. Resultantly, survival of patients with GC is usually awful on account of limited treatments [5]. Therefore, in order to develop effective therapeutic strategies to prolong the lifespan of GC patients, our priority is to understand the pathogenesis and detailed mechanisms underlying GC development.

Long non-coding RNAs (lncRNAs), a class of RNA transcripts belonging to non-coding RNAs family, are more than 200 nt in length and own limited protein-coding ability [6, 7]. Mounting evidence suggested that lncRNAs can serve crucial parts in different type of human cancers [8]. In recent decades, increasing lncRNAs have been unveiled to be involved in GC tumorigenesis [9]. For example, AK023391 contributes to tumorigenesis and invasion of GC via activating PI3K/Akt signaling pathway [10]. LINC00978 accelerates tumor growth in GC [11]. MALAT1 affects autophagy-associated chemoresistance in GC by sequestering miR-23b-3p [12]. TRERNA1 promotes the metastasis in GC by functioning as an enhancer of SNAI1 [13]. However, a vast majority of lncRNAs have never been explored in GC.

HOXC cluster antisense RNA 1 (HOXC-AS1) is a novel lncRNA which has never been investigated in cancer. Here, data from GSE109476 revealed that HOXC-AS1 was apparently highly-expressed in GC tissues compared to the normal tissues. In this basis, we wondered whether HOXC-AS1 was implicated in GC development. In the meantime, the in-depth mechanism whereby HOXC-AS1 elicited its function in GC was also focused on in the current study.

## Materials and methods

### Tissue specimen

35 paired GC tissues and adjacent normal tissues were attained from Quanzhou First Hospital Affiliated to Fujian Medical University. All patients had not been treated with any therapy before surgery. Written informed consents were offered by all participants. This study was permitted ethically by the ethics committee of Quanzhou First Hospital Affiliated to Fujian Medical University. Tissue samples were frozen in liquid nitrogen, storing at − 80 °C till RNAs were extracted.

### Microarray analysis

Differentially expressed genes from analyzing 5 matched human GC tissues and adjacent normal tissues were provided by GSE109476 and the results were exhibited as a heat map. *P* < 0.05 and fold change > 2 served as cut-off criteria.

### Cell culture

Normal stomach cell (GES-1), GC cells (MKN45, MKN28, MGC803, BGC-823 and AGS) and human embryonic kidney cell (HEK-293 T) were all purchased from the Shanghai Cell Bank of the Chinese Academy of Sciences (Shanghai, China). Cells were cultured as previously described [14–16].

### Cell transfection

BGC-823 or AGS cells were severally transfected with the plasmids mentioned below by utilizing Lipotransfectamine 3000 (Thermo Fisher Scientific, Waltham, MA, USA). Specific shRNAs against HOXC-AS1 (shHOXC-AS1#1 and shHOXC-AS1#2) or MYC (shMYC) and control (shCtrl), along with pcDNA3.1 vector containing HOXC-AS1 or MYC and empty vectors, were all from Genechem (Shanghai, China). MiR-590-3p mimics, miR-590-3p inhibitors and their corresponding miR-NCs were synthesized by GenePharma (Shanghai, China). Transfection lasted 48 h.

### Quantitative real-time PCR (qRT-PCR)

TRIzol reagent (Invitrogen, Carlsbad, CA, USA) was used to isolate total RNA. Complementary DNA (cDNA) was synthesized with PrimeScript RT Reagent Kit (TaKaRa, Osaka, Japan). Real-time PCR was carried out on IQ5 instrument (Bio-Rad, Hercules, CA, USA) applying SYBR Green fluorescence signal detection assays (TaKaRa). Gene expression levels were quantified through 2^-∆∆Ct^ method. U6 or GAPDH were the normalizations.

### Fluorescence in situ hybridization (FISH)

Design and synthesis of HOXC-AS1-FISH probe were accomplished by Invitrogen. BGC-823 or AGS cells were plated on culture slides, fixed in paraformaldehyde (PFA; Sigma-Aldrich, St. Louis, MO, USA), followed by the sealing with prehybridization buffer (Sigma-Aldrich). Hybridization mixture was added with FISH probe. Slides were washed in buffer adding saline sodium citrate (SSC; Sigma-Aldrich). Cell nuclei were stained by DAPI (Sigma-Aldrich). Cells were examined with Olympus fluorescence microscope (Olympus, Tokyo, Japan).

### Cell counting kit-8 (CCK-8) assay

Transfected cells in 96-well plates were subjected to 10 μl CCK-8 (TransGen Biotech, Beijing, China). Cell proliferation was assessed through measuring the OD value at 450 nm via microplate spectrophotometer (Bio-Tek, Winooski, VT, USA).

### EdU assay

Transfected BGC-823 or AGS cells were incubated with 50 μmol EdU (5-Ethynyl-2′-deoxyuridine) (Sigma-Aldrich), dyed in DAPI. EdU-positive cells were visualized with the fluorescence microscope.

### TUNEL assay

In situ cell death detection kit with horseradish peroxidase (POD; Roche, Basel, Switzerland) was utilized. Upon deparaffinization and rehydration, sections were treated with protease K (Invitrogen) and hydrogen peroxide (Sigma-Aldrich) to wipe off endogenous peroxidase. Samples were immersed in TUNEL (TdT-mediated dUTP Nick-End Labeling) reaction mixture, incubated with DAPI. Images were eventually captured via the fluorescence microscope.

### Transwell assay

Cell invasion and migration were tested with transwell insert chambers (Corning, NY, USA) with or without Matrigel (BD, NJ, USA). Transfected BGC-823 or AGS cells were added to the upper chamber with serum-free medium. 20% FBS-containing medium was placed to the bottom chamber. 48 h later, cells on the bottom were fixed and dyed utilizing crystal violet (Sigma-Aldrich). Cells were eventually counted in at least three randomly picked microscopic fields.

### Chromatin Immunoprecipitation (ChIP)

An EZ-Magna ChIP kit (Millipore) was employed to conduct ChIP assay as described in previous study [17]. Antibodies against c-MYC (Abcam, Cambridge, USA) and IgG (Abcam) were individually applied.

### Western blot

Western blot were carried out as previously described [18]. Primary antibodies against E-cadherin (ab40772), N-cadherin (ab76057), Vimentin (ab8978), β-catenin (ab16051), CBP (ab50702), BRG1 (ab108318), MYC (ab9106) and GAPDH (ab245356) and secondary antibodies were all obtained from Abcam.

### Luciferase reporter assay

The pGL3-HOXC-AS1 promoter WT/MUT was co-transfected into cells with shMYC or pcDNA3.1/MYC or shCtrl or pcDNA3.1. The pGL3-MYC promoter was co-transfected into BGC-823 or AGS cells with shHOXC-AS1#1 or shCtrl. Using pmirGLO dual-luciferase plasmid (Promega, Madison, WI, USA), HOXC-AS1-WT/MUT or MYC-WT/MUT were constructed and co-transfected with indicated transfection plasmids. Analysis was conducted via dual-luciferase reporter assay system (Promega).

### RNA Immunoprecipitation (RIP)

A Magna RIP™ RNA-Binding Protein Immunoprecipitation Kit (Millipore, Bedford, MA, USA) was applied. Antibodies against Ago2 (Abcam), CBP, BRG1 and IgG were adopted for RIP assay.

### DNA pull-down assay

Cell lysates from BGC-823 or AGS cells were incubated with HOXC-AS1 biotin probe or HOXC-AS1 no-biotin probe, followed by incubation with streptavidin agarose beads (Life Technologies, Gaithersburg, MD, USA). Finally, the captured DNAs were reversely transcribed into cDNA and then determined by qRT-PCR.

### Co-immunoprecipitation (CoIP) assay

The interacting proteins remained in cell lysates were co-precipitated using the specific antibodies against BRG1 and β-catenin (both from Cell Signaling Technology, Boston, MA, USA). Then target protein was drawn down in accordance with electrophoresis bands and analyzed finally with western blotting.

### TOP/FOP flash assay

BGC-823 or AGS cells were transfected with TOP Flash or FOP Flash (Upstate Biotechnology, Lake Placid, NY, USA). The medium was then exchanged to a medium containing shHOXC-AS1#1 or shCtrl. Luciferase assay was conducted after cells were lysed.

### In vivo growth and metastasis experiments

As for the in vivo tumor growth assays, AGS cells transfected with shHOXC-AS1#1 or shCtrl were subcutaneously injected into BALB/c athymic nude mice (4 weeks old) that were bought from Nanjing University (Nanjing, China). Tumors volume was recorded every 4 days for 4 weeks and tumors weight was measured after mice were killed. With respect to the in vivo metastatic experiments, shCtrl- or shHOXC-AS1#1-transfected AGS cells were injected into the tail vein of nude mice. Eight weeks later, the mice were sacrificed and metastatic nodules in livers were calculated and photographed. Besides, all the tumors were processed for hematoxylin-eosin (HE) staining or immunohistochemistry (IHC) staining, as appropriate. All experimental procedures were approved by Animal Care and Use Committee of Quanzhou First Hospital Affiliated to Fujian Medical University.

### In-situ hybridization (ISH) assay

The expression of HOXC-AS1 in the paraffin-embedded sections of above tumors was assessed via ISH assay according to the previous report [19].

### Immunohistochemistry (IHC) staining

In line with previous protocol [20], IHC staining was performed with the use of primary antibodies against c-MYC, Ki-67, E-cadherin and N-cadherin (all from Abcam).

### Statistical analysis

Experiments were conducted for thrice. Data were determined as mean ± SD and assayed via GraphPad Prism 7.0 (GraphPad Software, La Jolla, CA, USA) and SPSS 23.0 (IBM, Armonk, NY, USA). ANOVA or Student’s t-test was used for difference analysis. *P* < 0.05 indicated statistically significant.

## Results

### Overexpressed HOXC-AS1 in GC locates both in the cytoplasm and nucleus of GC cells

**Fig. 1:**
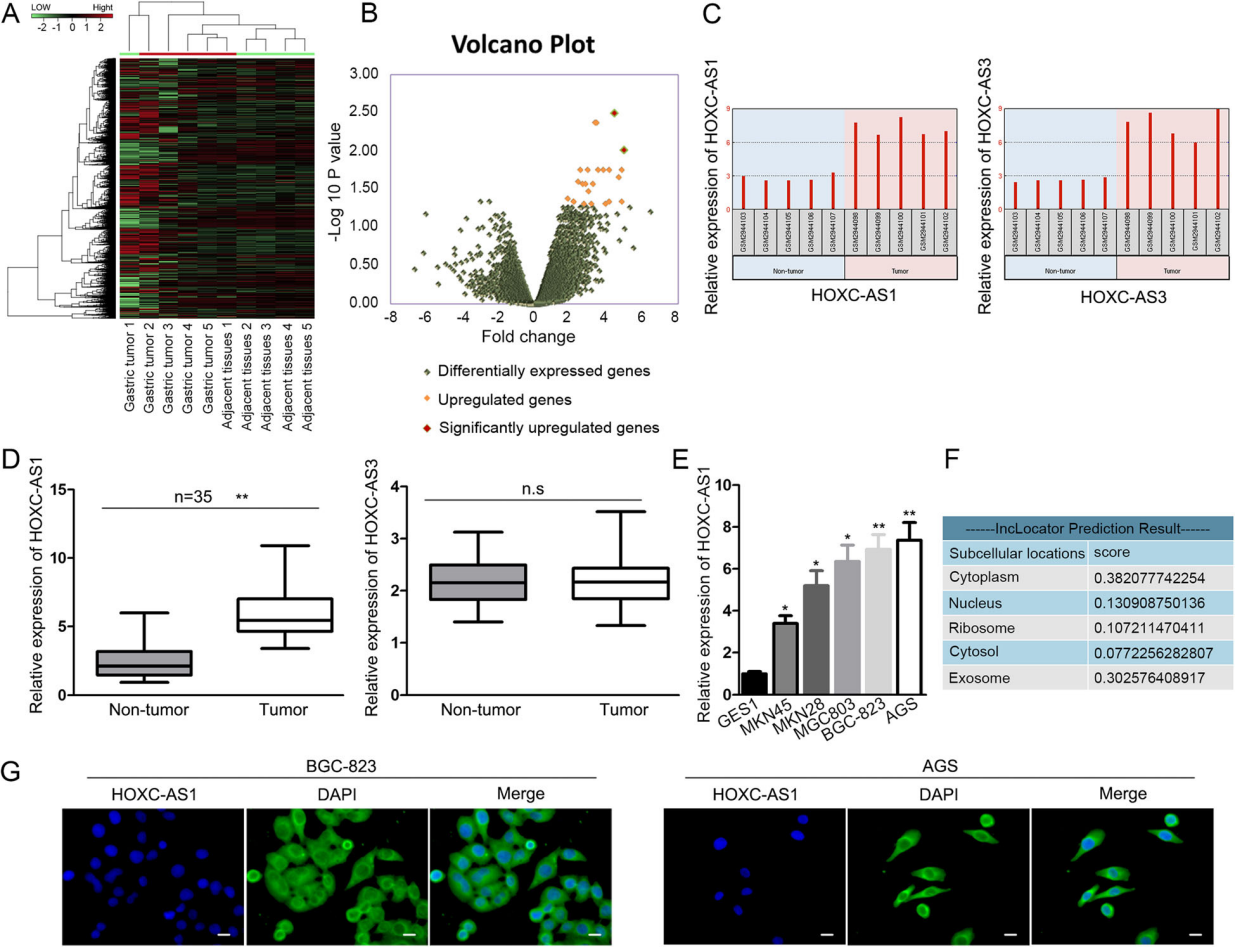
HOXC-AS1 was overexpressed in GC tissues and cell lines. (**a, b**) Heat map and volcano plot obtained from analyzing GSE109476. (**c**) GSE109476 suggested that both HOXC-AS1 and HOXC-AS3 was highly-expressed in GC tissues compared to the adjacent ones. (**d**) qRT-PCR result of HOXC-AS1 and HOXC-AS3 in 35 pairs of GC tissues collected in our study. (**e**) The expression of HOXC-AS1 in GC cell lines was examined by qRT-PCR. (**f**) The potential localization of HOXC-AS1 predicted by lncLocator. (**g**) FISH analysis of HOXC-AS1 location in GC cells. * *P* < 0.05, ** *P* < 0.01

To recognize the lncRNAs implicated in the development of GC, we analyzed the expression profile of dysregulated mRNAs and lncRNAs in GC tissues and adjacent normal tissues from GEO dataset numbered GSE109476. As displayed in Fig. 1a, over 20,000 lncRNAs and protein-coding genes were dysregulated in GC tissues in comparison to adjacent normal tissues. Furtherly, among all the differentially expressed genes (fold change > 2, *P* < 0.05), two lncRNAs, HOXC-AS1 and HOXC-AS3, were the most upregulated lncRNAs with the fold change > 16 (*P* < 0.01) (Fig. 1b). Besides, data obtained from GSE109476 suggested the markedly enhanced expression level of HOXC-AS1 and HOXC-AS3 in GC tissues compared to adjacent non-tumor tissues (Fig. 1c). Next, we detected the level of above two lncRNAs in another 35 pairs of GC tissues obtained in our study. As a result, it was indicated that only HOXC-AS1 was obviously upregulated in GC tissues relative to the para-carcinoma tissues, whereas no significant enhancement of HOXC-AS3 expression was observed in GC tissues in contrast to adjacent non-cancerous tissues (Fig. 1d). Moreover, the high expression of HOXC-AS1 was confirmed in five GC cell lines (MKN45, MKN28, MGC803, BGC-823 and AGS) compared to the normal GES-1 cells (Fig. 1e). In addition, prediction of bioinformatics tool lncLocator showed that HOXC-AS1 was distributed abundantly in cytoplasm, exosome, nucleus, and ribosome (Fig. 1f). Accordingly, FISH staining confirmed the fluorescence intensity of HOXC-AS1 in both cytoplasm and nucleus of two GC cells that expressed highest HOXC-AS1 endogenously (Fig. 1g). Based on these data, we speculated that HOXC-AS1 might play a role in GC development.

### Loss of HOXC-AS1 function hinders the proliferation, motility and EMT in GC cells

**Fig. 2:**
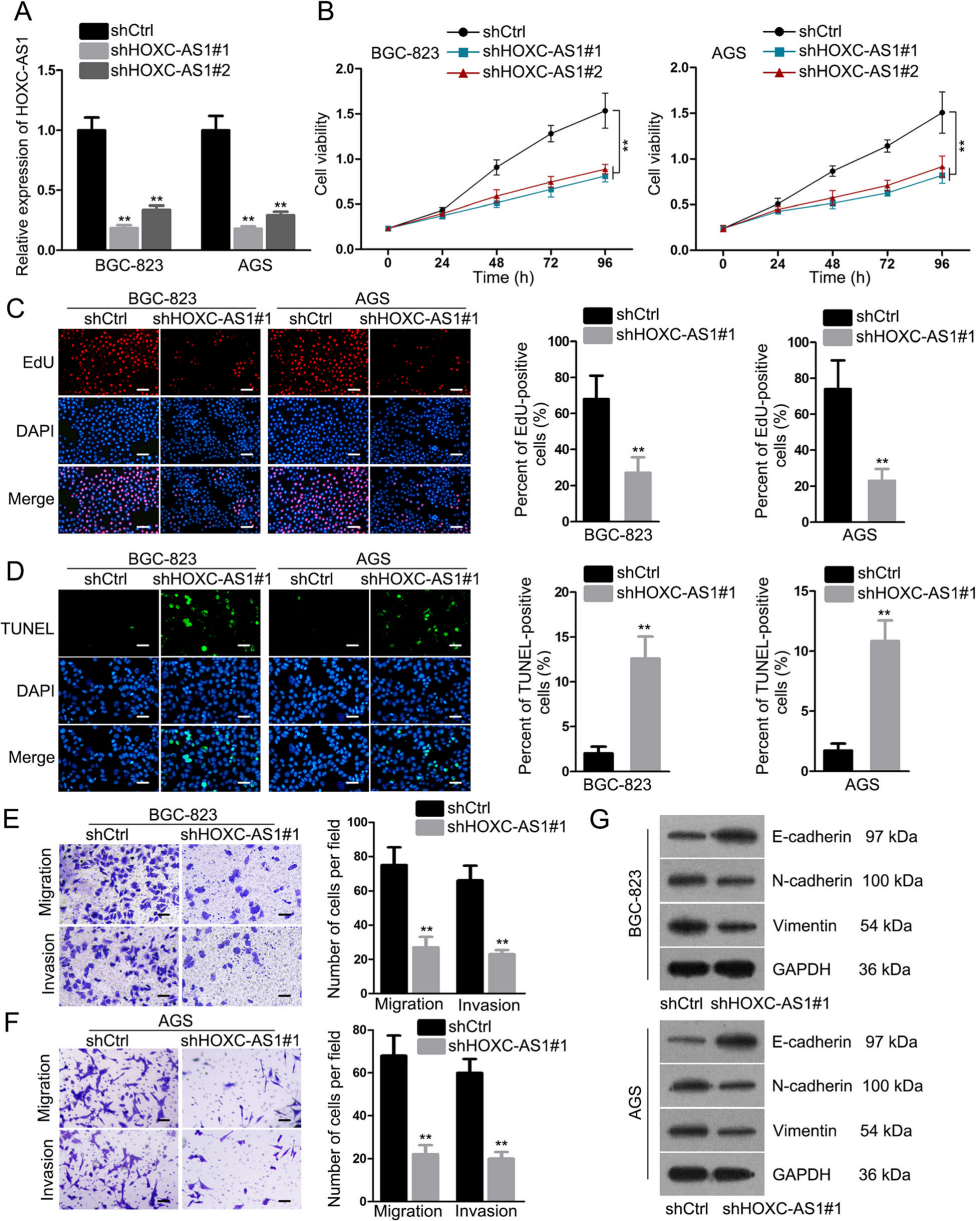
Knockdown of HOXC-AS1 inhibited cell proliferation, migration, invasion and EMT in GC. (**a**) qRT-PCR result of HOXC-AS1 in BGC-823 and AGS cells transfected with shCtrl or two shRNAs aiming at HOXC-AS1. (**b**) The viability of BGC-823 and AGS cells under above transfections was assessed by CCK-8 assays. (**c, d**) EdU and TUNEL assays were respectively performed to evaluate cell proliferation and apoptosis in BGC-823 and AGS cells with HOXC-AS1 inhibition or not. (**e, f**) Transwell assays were implemented to estimate the effect of HOXC-AS1 on GC cell migration and invasion. Western blot analysis of E-cadherin, N-cadherin and Vimentin in BGC-823 and AGS under HOXC-AS1 knockdown versus control. ** *P* < 0.01

In order to confirm the function of HOXC-AS1 in GC, we then detected its influence on the biological processes in vitro. As revealed by qRT-PCR analysis, the expression of HOXC-AS1 was largely restrained in both BGC-823 and AGS cells after being transfected with two shRNAs against HOXC-AS1 (Fig. 2a). Resultantly, the viability of above two cells was also suppressed under HOXC-AS1 inhibition (Fig. 2b). Besides, cells transfected with shHOXC-AS1#1 was further used in subsequent experiments due to the higher silencing efficiency. Consequently, the result of EdU assay indicated that knockdown of HOXC-AS1 led to lessened proliferation in both BGC-823 and AGS cells (Fig. 2c), and that of TUNEL assay revealed that HOXC-AS1 silence distinctly stimulated GC cell apoptosis (Fig. 2d). Moreover, we also proved that the migratory and invasive abilities of both BGC-823 and AGS cells were weakened in response to HOXC-AS1 inhibition (Fig. 2e-f). Similarly, depletion of HOXC-AS1 also apparently repressed epithelial-mesenchymal transition (EMT) in GC cells, as the level of epithelial marker E-cadherin was heightened while that of the mesenchymal markers N-cadherin and Vimentin lowered in the context of HOXC-AS1 suppression (Fig. 2g). By and large, HOXC-AS1 plays a facilitating role in GC development.

### HOXC-AS1 is transcriptionally activated by c-MYC in GC

**Fig. 3:**
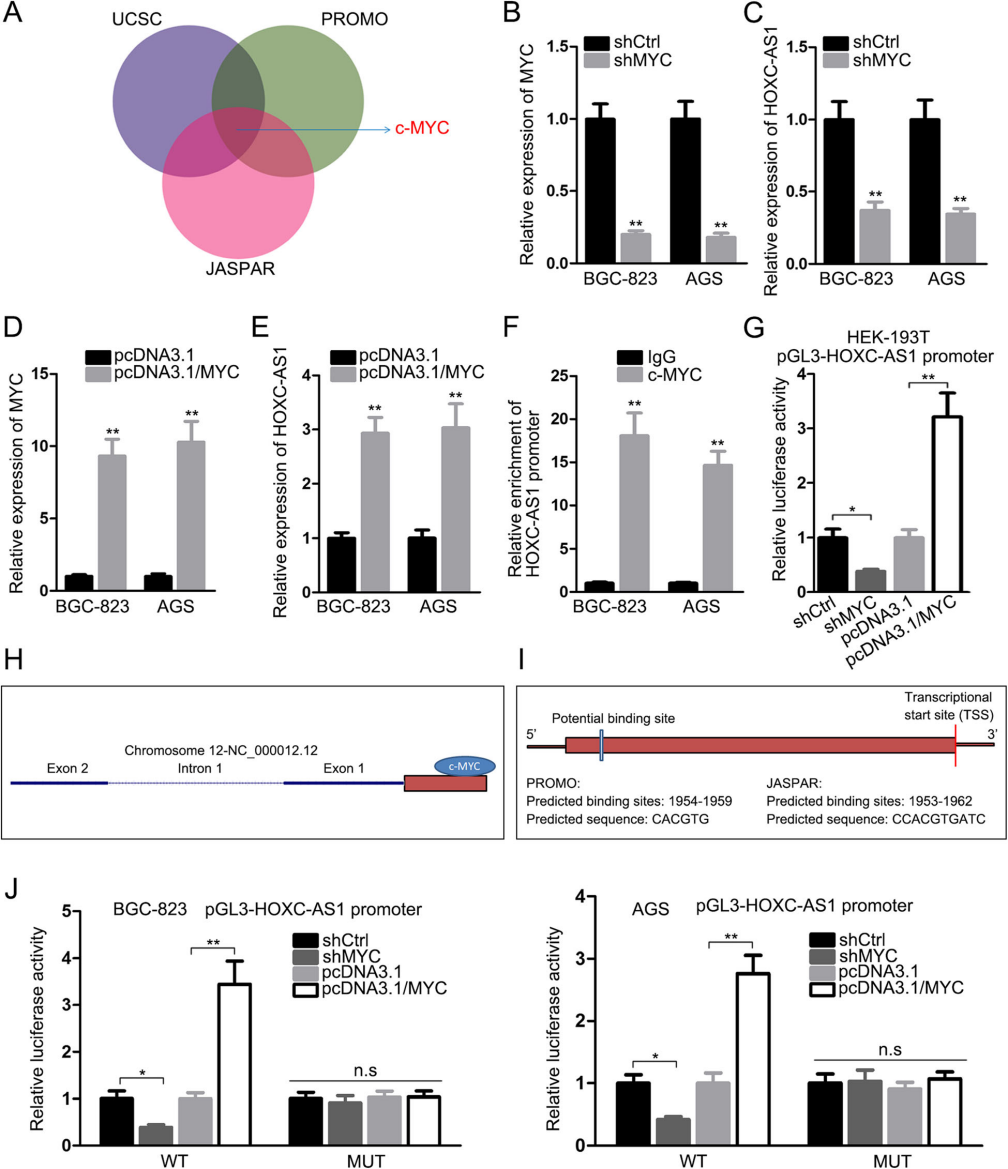
HOXC-AS1 was transcriptionally upregulated by c-MYC in GC. (**a**) Three online tools including UCSC, JASPAR and PROMO predicted that c-MYC might potentially regulate HOXC-AS1 transcription. (**b, e**) The expression of MYC and HOXC-AS1 in GC cells with MYC downregulation or overexpression was assayed by qRT-PCR. (**f, g**) ChIP and luciferase reporter assays revealed that HOXC-AS1 was positively regulated by c-MYC at transcriptional level. (**h**, **i**) The predicted binding of c-MYC on HOXC-AS1 promoter region was simulated here. (**j**) Luciferase reporter assay confirmed the pricise binding of c-MYC to HOXC-AS1 promoter at site from − 1954 to − 1959. * *P* < 0.05, ** *P* < 0.01

Subsequently, we wanted to know how HOXC-AS1 was upregulated in GC. As predicted by three online tools including UCSC, JASPAR and PROMO, HOXC-AS1 seemed to be regulated by c-MYC, a well-recognized oncogene in diverse cancers including GC (Fig. 3a). In addition, we found that HOXC-AS1 was positively regulated by MYC, evidenced by HOXC-AS1 expression in two GC cells was reduced after silencing MYC but enhanced when overexpressing MYC (Fig. 3b-e). Moreover, the ChIP assay verified a predominant enrichment of HOXC-AS1 promoter in c-MYC-binding compounds (Fig. 3f). Of note, the luciferase activity of pGL3-HOXC-AS1 promoter was dampened by suppressing MYC but strengthened by overexpressing MYC (Fig. 3g). Subsequently, we found that the putative binding sites of c-MYC to HOXC-AS1 promoter region predicted by PROMO (− 1954 to − 1959) and JASPAR (− 1953 to − 1962) were overlapped (Fig. 3h, i). In this basis, the sequences of HOXC-AS1 promoter from − 1954 to − 1959 were mutated to further confirm the specific binding of c-MYC to HOXC-AS1 promoter. As expected, neither upregulation nor downregulation of MYC affected the luciferase activity of pGL3-HOXC-AS1 promoter with the mutation of predicted MYC sites in both BGC-823 and AGS cells (Fig. 3j), suggesting the precise interaction of c-MYC with HOXC-AS1 promoter at the sequences from − 1954 to − 1959. According to these findings, we came into a conclusion that HOXC-AS1 is trans-activated by c-MYC in GC.

### Cytoplasmic HOXC-AS1 interacts with miR-590-3p to upregulate MYC expression in turn

**Fig. 4:**
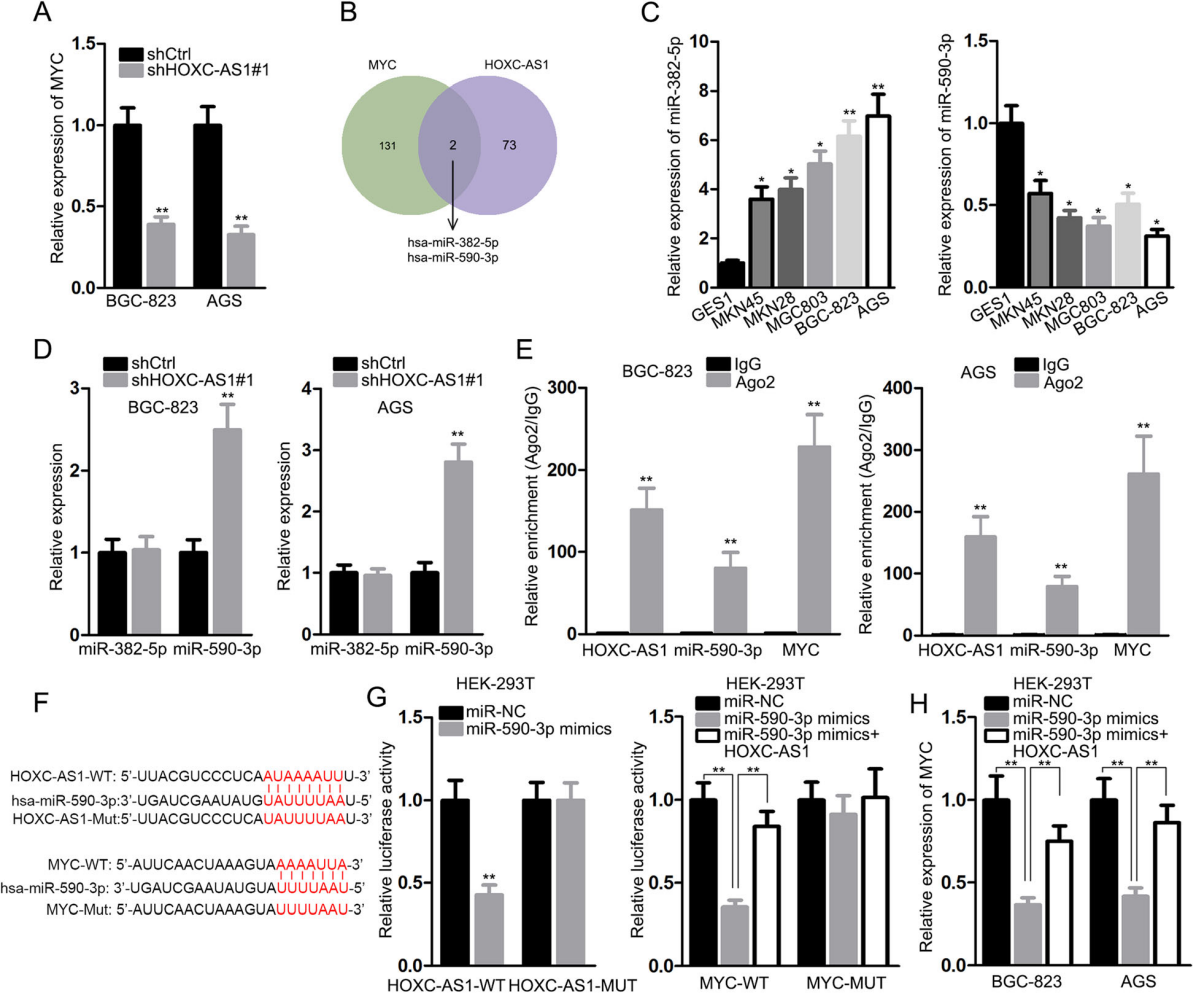
HOXC-AS1 triggered MYC expression in GC by absorbing miR-590-3p in cytoplasm. (**a**) qRT-PCR result of MYC level in GC cells with or without HOXC-AS1 silence. (**b**) DIANA predicted that there was only two miRNA that interacted with both HOXC-AS1 and MYC. (**c**) The expression levels of above two miRNAs in GC cell lines were determined by qRT-PCR. (**d**) qRT-PCR result of the two miRNAs in BGC-823 and AGS cells with HOXC-AS1 inhibition or not. (**e**) RIP assay proved the co-existence of HOXC-AS1, miR-590-3p and MYC mRNA in RISC. (**f, g**) Luciferase reporter assay showed the competition binding of HOXC-AS1 and MYC mRNA to miR-590-3p. (H) Relative expression of MYC in indicated BGC-823 and AGS cells was analyzed via qRT-PCR. * *P* < 0.05, ** *P* < 0.01

Considering the tumorigenic role of c-MYC in diverse cancers including GC, we wondered whether HOXC-AS1 could regulate MYC expression in GC cells. As proved in Fig. 4a, the level of MYC in both BGC-823 and AGS cells was markedly restrained by HOXC-AS1 depletion. In view of most HOXC-AS1 distributed in the cytoplasm, we were curious about whether it functioned as a competing endogenous RNA (ceRNA) to affect gene expressions at post-transcriptional level via sponging miRNAs [21]. Fortunately, we unveiled that 2 miRNAs, miR-382-5p and miR-590-3p, were predicted by DIANA to interact with both HOXC-AS1 and MYC mRNA (Fig. 4b). However, it was examined that the expression level of miR-382-5p was upregulated in GC cell lines compared with the normal GES-1 cells, whereas that of miR-590-3p was remarkably downregulated in GC cell lines relative to GES-1 cells (Fig. 4c). Besides, we observed that only miR-590-3p, but not miR-382-5p, was affected by HOXC-AS1 in GC, since the level of miR-590-3p was increased while the miR-382-5p level unchanged under HOXC-AS1 knockdown (Fig. 4d). Thus, we suspected that miR-590-3p was involved in ceRNA network regarding HOXC-AS1 and MYC mRNA.

Thereafter, we detected an obvious co-harvest of HOXC-AS1, miR-590-3p and MYC mRNA in the complex immunoprecipitated by anti-Ago2 (Fig. 4e), implying the co-existence of these three RNAs in RNA-induced silencing complex (RISC). Moreover, the competition between HOXC-AS1 and MYC mRNA in interacting with miR-590-3p was further validated by luciferase reporter assay. Results presented that ectopic expression of miR-590-3p controlled the luciferase activity of both HOXC-AS1-WT and MYC-WT and its inhibition on MYC-WT luciferase activity was attenuated under HOXC-AS1 overexpression (Fig. 4f, g). Accordantly, MYC level that was impeded by miR-590-3p upregulation was also recovered in face of HOXC-AS1 overexpression (Fig. 4h). To sum up, HOXC-AS1 boosts MYC mRNA expression in the cytoplasm of GC cells via sponging miR-590-3p.

### HOXC-AS1 promotes MYC transcription via enhancing the interaction of BRG1 with β-catenin in the nucleus of GC cells

**Fig. 5:**
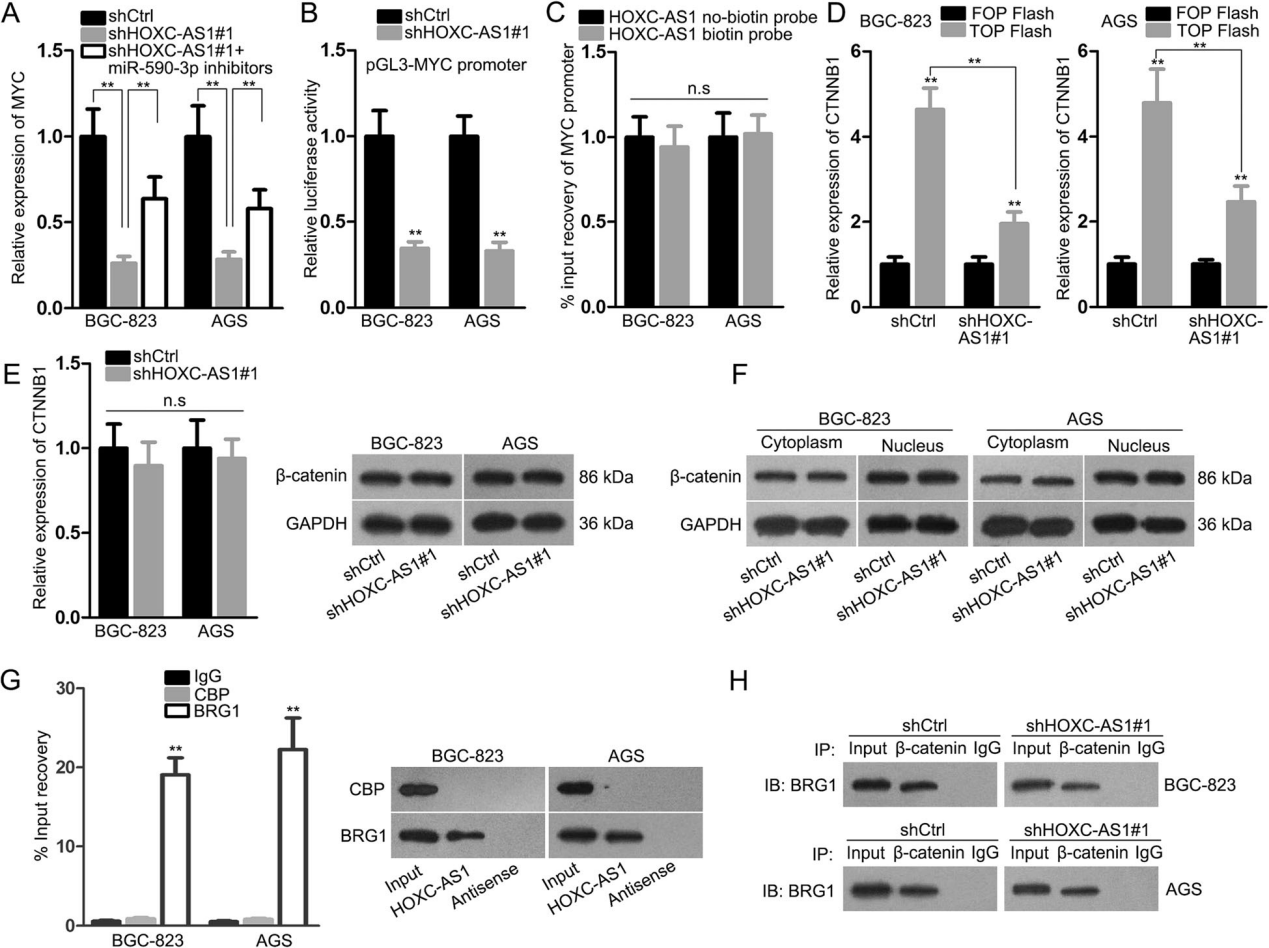
HOXC-AS1 promoted BRG1 interaction with β-catenin to enhance MYC transcription. (**a**) qRT-PCR result of the expression of MYC in BGC-823 and AGS cells in response to HOXC-AS1 silence or not, or HOXC-AS1 silence plus miR-590-3p inhibition. (**b**) The effect of HOXC-AS1 on MYC transcription was assessed by luciferase reporter assay. (**c**) The binding of HOXC-AS1 to MYC promoter was examined by DNA pull down assay. (**d**) TOP/FOP flash assay was conducted to estimate the impact of HOXC-AS1 on Wnt/β-catenin activation. (**e, f**) The influence of HOXC-AS1 on CTNNB1 expression and β-catenin nuclear translocation was determined by qRT-PCR and western blot, as appropriate. (**g**) The interaction between HOXC-AS1 and BRG1 in GC cells was testified by RIP and RNA pull down assays. (**h**) The interaction between BRG1 and β-catenin in GC cells with or without HOXC-AS1 knockdown was evaluated by CoIP assay. ** *P* < 0.01

Interestingly, it seemed that HOXC-AS1 affected MYC expression in GC through not only miR-590-3p-mediated manner but also another unknown pathway because the qRT-PCR revealed that miR-590-3p inhibitor only partly restored MYC level in GC cells transfected with shHOXC-AS1#1 (Fig. 5a). Previously, we found that HOXC-AS1 was located not only in cytoplasm, but also in nucleus. Hence, we wondered whether HOXC-AS1 had an impact on MYC transcription. As a result, the luciferase activity of pGL3-MYC promoter was hampered upon HOXC-AS1 inhibition, whereas no direct binding of HOXC-AS1 with MYC promoter was captured (Fig. 5b, c), indicating that HOXC-AS1 regulated MYC transcription without interacting with its promoter. It is widely known that the activation of Wnt/β-catenin pathway leads to the transactivation of many downstream effectors including MYC [22]. Wnt/β-catenin pathway is a classic signaling that regulating cell growth and metastasis in a wide range of cancer types [23, 24], including in GC [25]. Therefore, we wondered whether HOXC-AS1 regulated MYC transcription through Wnt/β-catenin pathway. TOP/FOP flash assay revealed that the activity of Wnt/β-catenin signaling was noticeably confined when inhibiting HOXC-AS1 (Fig. 5d). In addition, neither the expression of CTTNB1 mRNA and β-catenin protein nor the nuclear translocation of β-catenin protein was affected by HOXC-AS1 depletion (Fig. 5e, f).

Considering the transcriptional activity of TCFs was affected by not only β-catenin but also β-catenin-recruited co-activators [26], we assumed that HOXC-AS1 might influence the transcription of TCF4 targets through affecting transcriptional co-activators in this situation. BRG1 and p300/CBP were known co-activators that were recruited by β-catenin to the promoter of TCF targets [27]. Meanwhile, the RIP-Seq analysis demonstrated that HOXC-AS1 interacted with BRG1 rather than CBP. Furthermore, it was testified that HOXC-AS1 interacted with BRG1 but not CBP in GC cells (Fig. 5g). More importantly, the CoIP result validated that the interaction between BRG1 and β-catenin was hampered in GC cells facing HOXC-AS1 depletion (Fig. 5h). Collectively, these findings unveiled that HOXC-AS1 evokes MYC transcription by strengthening the binding of BRG1-β-catenin-TCF4 complex to MYC promoter.

### HOXC-AS1 depletion dampens GC tumor growth and metastasis through MYC in vivo

**Fig. 6:**
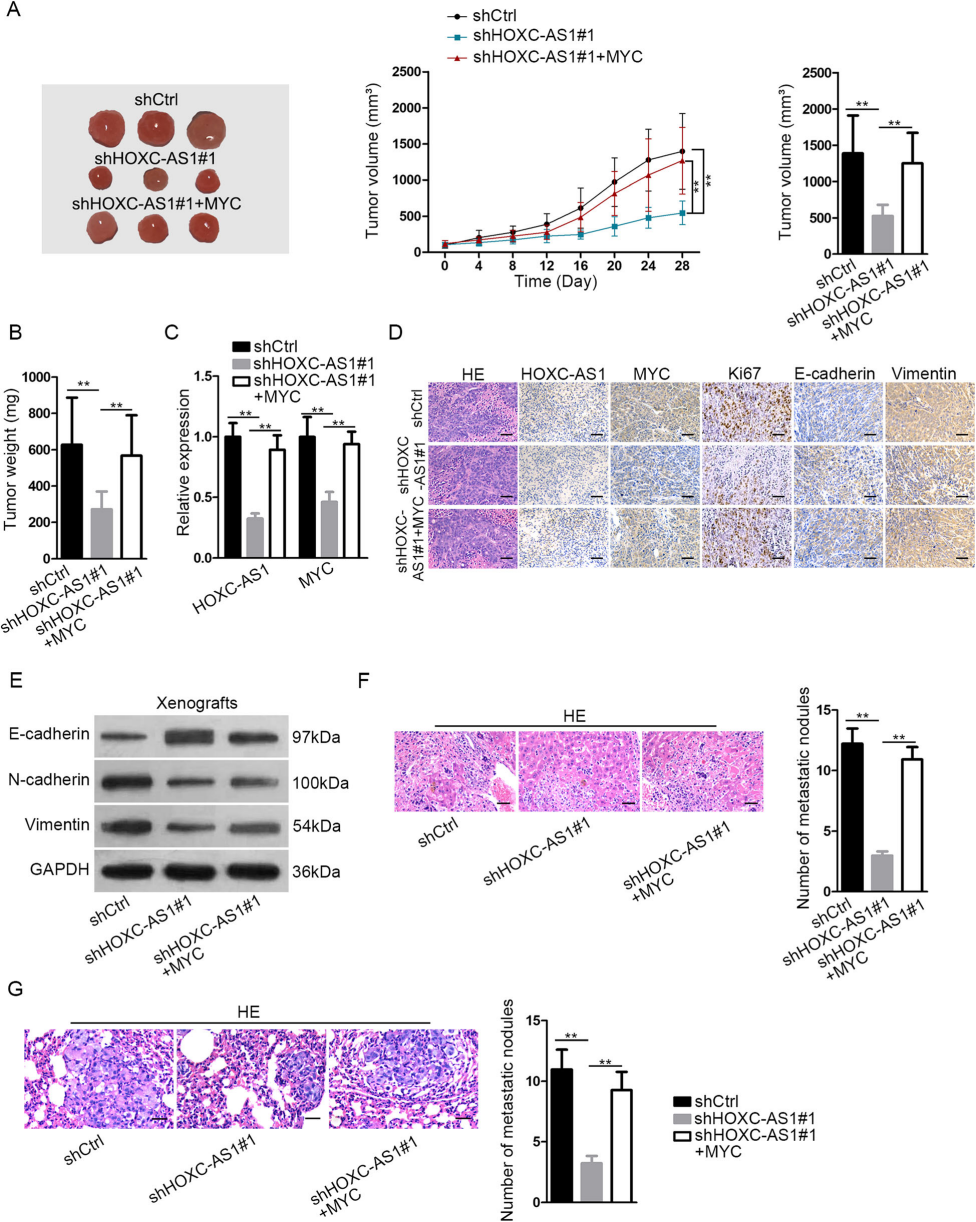
Silencing HOXC-AS1 confined GC tumorigenesis and metastasis in vivo. (**a**) Representative images and tumor volume of AGS cells transfected with shCtrl, shHOXC-AS1, or shHOXC-AS1 + pcDNA3.1/MYC. (**b**) Mean weight of these tumors. (**c**) qRT-PCR result of the expression of HOXC-AS1 and MYC in above tumors. (**d**) The expression of HOXC-AS1, MYC, Ki67, E-cadherin and N-cadherin in those tumors was tested by ISH or IHC staining, as needed. (**e**) The level of EMT-related proteins was determined by western blot. (**f, g**) HE staining of livers and lung obtained from in vivo metastatic experiments and quantitation of the metastatic nodules in these livers. ** *P* < 0.01

In order to further certify the function of HOXC-AS1/MYC axis in GC progression, AGS cells transfected with shCtrl, shHOXC-AS1#1, or shHOXC-AS1#1 + MYC were introduced into nude mice and the growth of GC cells in vivo was monitored. As indicated in Fig. 6a, the tumors derived from HOXC-AS1-silenced AGS cells were smaller in size and grew slower than those from shCtrl-transfected control cells, and overexpression of MYC recovered the tumor size and tumor growth. Consistently, the reduced tumor weight under HOXC-AS1 suppression was restored by overexpression of MYC in vivo (Fig. 6b). Of importance, we revealed that HOXC-AS1 and MYC mRNA levels decreased upon the knockdown of HOXC-AS1 in xenografts and such decrease was reversed by MYC overexpression (Fig. 6c). Moreover, ISH staining of HOXC-AS1 and IHC staining of MYC, Ki67, N-cadherin, and Vimentin decreased, whereas IHC staining of E-cadherin increased under HOXC-AS1 silence in vivo, and these results were reversed by the overexpression of MYC (Fig. 6d). Besides, upregulation of E-cadherin protein and downregulation of N-cadherin and Vimentin proteins were also observed in tumors with HOXC-AS1 depletion, and such phenomenon was reversed by the forced expression of MYC in vivo (Fig. 6e). Furtherly, the result of in vivo metastatic experiments indicated that silencing HOXC-AS1 remarkably lessened the secondary metastatic nodules in the livers and lungs of mice, and such effect was abrogated by the ectopic expression of MYC (Fig. 6f, g). According to these data, we concluded that knockdown of HOXC-AS1 suppresses GC cell growth and metastasis through MYC in vivo.

### Enforced expression of MYC reverses HOXC-AS1 silence-repressed malignant phenotypes in GC cells

**Fig. 7:**
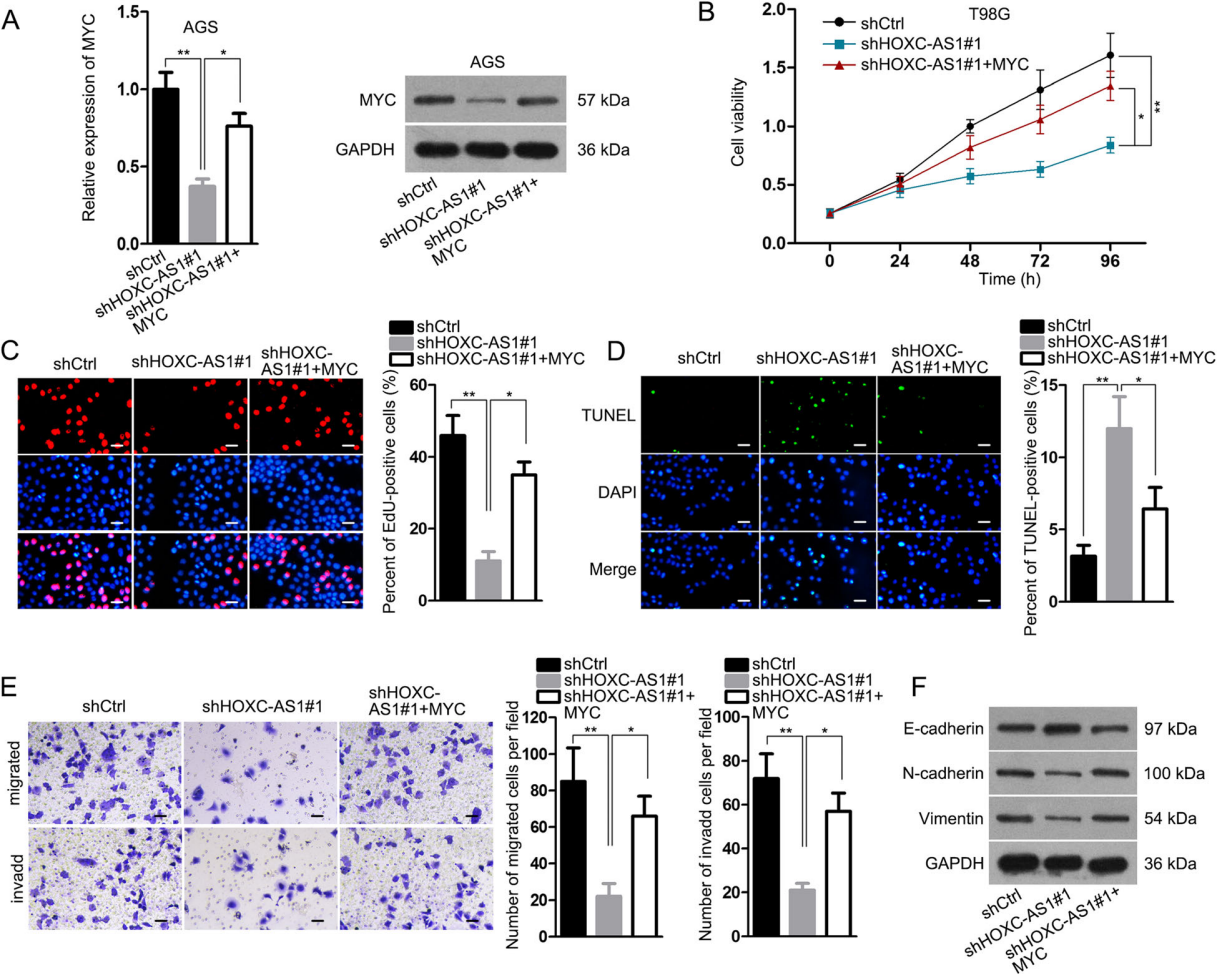
MYC upregulation reversed the suppression of HOXC-AS1 inhibition on the biological processes of GC cells. (**a**) qRT-PCR and western blot analyses were performed to assay the expression of MYC at both mRNA and protein levels in AGS cells transfected with shCtrl, shHOXC-AS1#1 or shHOXC-AS1#1 together with pcDNA3.1 plasmid containing MYC. (**b-e**) The viability, proliferation, apoptosis and motility in above AGS cells were assessed respectively by CCK-8, EdU, TUNEL and transwell assays. (**f**) The level of EMT-associated proteins in indicated AGS cells was determined by western blot. * *P* < 0.05, ** *P* < 0.01

Subsequently, we attempted to validate in vitro whether MYC was responsible for HOXC-AS1-contributed GC development. Firstly, it was confirmed that the reduced expression of MYC at both mRNA and protein levels in HOXC-AS1-depleted AGS cells was normalized under the co-transfection of pcDNA3.1/MYC (Fig. 7a). As a consequence, the repressing effect of shHOXC-AS1#1 on viability and proliferation in AGS cells were impaired in face of MYC upregulation (Fig. 7b, c), while an opposite performance was observed in the apoptosis of AGS cells facing equal conditions (Fig. 7d). Moreover, it was suggested that ectopic expression of MYC obviously counteracted the inhibition of HOXC-AS1 knockdown on GC cell migration and invasion (Fig. 7e). Likewise, upregulated MYC also led to a definite recovery on the EMT process that was hindered by HOXC-AS1 depletion (Fig. 7f). Altogether, these results unveiled that HOXC-AS1 exerts its promoting function in GC development via a MYC-dependent manner.

## Discussion

In the past few decades, lncRNAs have emerged as new regulators in the initiation and progression of numerous human cancers [28, 29], including GC [30–32]. In the present study, we firstly figured out a novel lncRNA HOXC-AS1 which had never been explored in cancer before. Previous study by Huang et al. only reported the suppressive role of HOXC-AS1 in ox-LDL-induced cholesterol accumulation [33]. Besides, it was discovered that HOXC-AS1 was highly expressed in GC specimens and cell lines, and was distributed in both the cytoplasm and nucleus of GC cells. Functionally, knocking down HOXC-AS1 hampered GC cell growth and metastasis both in vitro and in vivo.

In subsequence, we found that HOXC-AS1 could be transcriptionally activated by c-MYC, a proto-oncogene that encodes a nuclear phosphoprotein which participates in diverse cellular processes, such as cell cycle progression, proliferation, apoptosis, migration, and EMT [34]. In addition, the contribution of MYC to the carcinogenesis of GC has also been uncovered previously [35, 36]. However, the link between MYC and HOXC-AS1 has never been established before. Intriguingly, our study also proved that HOXC-AS1 in turn had a regulatory effect on MYC expression through two different pathways. On one hand, consistent with the known knowledge that cytoplasmic lncRNAs as a ceRNA in modulating cancer development by affecting protein-coding genes at post-transcriptional level via secluding miRNAs [37, 38], we firstly revealed that cytoplasmic HOXC-AS1 acted as a ceRNA of MYC mRNA in GC cells through competitively interacting with miR-590-3p. Formerly, studies depicted that miR-590-3p exerted repressing impacts in nasopharyngeal carcinoma, cervical cancer, breast cancer, and gastric cancer [39–42]. Concordantly, our findings suggested that miR-590-3p served as a negative regulator in GC. On the other hand, we firstly showed that HOXC-AS1 transcriptionally induced MYC in nucleus, because our data suggested that miR-590-3p only mediate the regulatory function of HOXC-AS1 in GC partly, which meant that HOXC-AS1 regulated MYC through other manners. As largely reported, the activation of Wnt/β -catenin pathway stimulate the transcription of MYC [22], and influence growth and metastasis in cancer cells [23, 24]. We discovered that HOXC-AS1 activated Wnt/β-catenin signaling in GC. Furtherly, our study firstly showed that HOXC-AS1 in the nucleus of GC cells allowed BRG1 interacting with β-catenin to activate MYC transcription, while similar mechanism by which nuclear lncRNAs affect gene expressions has already been demonstrated over past years [43–45]. Besides, the interaction of BRG1 with β-catenin has been validated by a previous study conducted by Barker et al. [27], but we firstly showed that the BRG1-β-catenin interaction could be enhanced by HOXC-AS1. Last but not least, we demonstrated that MYC was the terminal effector responsible for HOXC-AS1-facilitated GC development.

## Conclusion

**Fig. 8:**
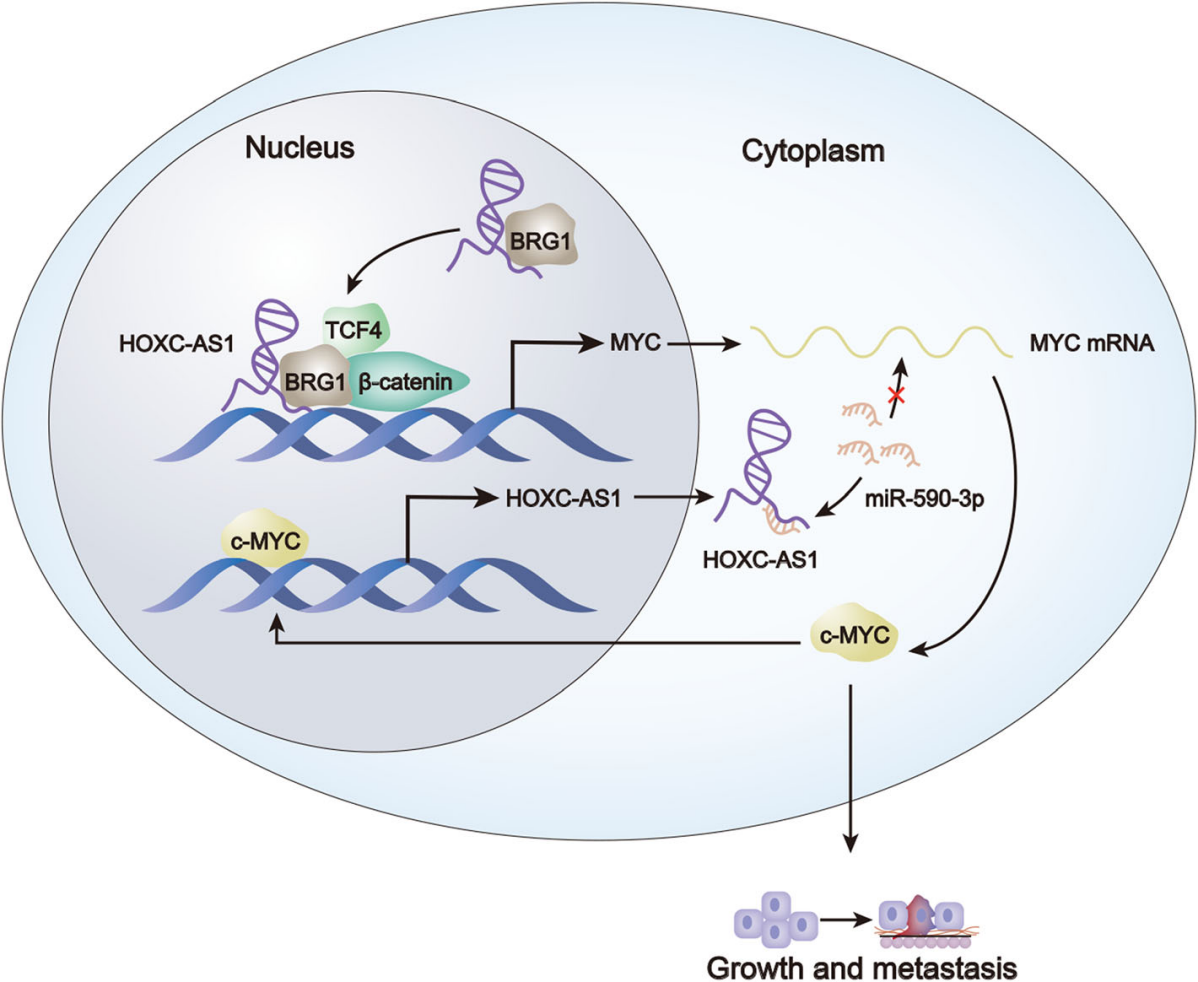
Schematic model of the HOXC-AS1-MYC feedback loop in aggravating GC tumorigenesis and metastasis. C-MYC-activated HOXC-AS1 sponges miR-590-3p in cytoplasm to stabilize MYC mRNA and strengthens BRG1-β-catenin interaction to promote MYC transcription in nucleus in the meantime, leading to prompted MYC expression and resultantly accelerated GC progression

In summary, the present research elucidated a HOXC-AS1-MYC feed-forward loop in exacerbating tumor growth and metastasis in GC (Fig. 8), which offers the first evidence for HOXC-AS1 as a tumorigenic lncRNA in cancer and could also highlight HOXC-AS1 as a promising target for GC treatment. Nevertheless, more proofs need to be dug out in the future to further strengthen the clinical significance of HOXC-AS1 in GC or even other human cancers.

## Abbreviation

CCK-8: Cell counting kit-8
ceRNA: Competing endogenous RNA
ChIP: Chromatin Immunoprecipitation
CoIP: Co-immunoprecipitation
EdU: 5-Ethynyl-2′-deoxyuridine
EMT: Epithelial-mesenchymal transition
FISH: Fluorescence in situ hybridization
GC: Gastric cancer
HE: Hematoxylin-eosin
HOXC-AS1: HOXC cluster antisense RNA 1
IHC: Immunohistochemistry
ISH: In-situ Hybridization
lncRNAs: Long non-coding RNAs
qRT-PCR: Quantitative real-time PCR
RIP: RNA Immunoprecipitation
RISC: RNA-induced silencing complex
TUNEL: TdT-mediated dUTP Nick-End Labeling

## References

[1] McLean MH, El-Omar EM (2014). Genetics of gastric cancer. Nat Rev Gastroenterol Hepatol.

[2] Torre LA, Bray F, Siegel RL, Ferlay J, Lortet-Tieulent J, Jemal A (2015). Global cancer statistics, 2012. CA Cancer J Clin.

[3] Dassen AE, Dikken JL, van de Velde CJH, Wouters MWJM, Bosscha K, Lemmens VEPP (2013). Changes in treatment patterns and their influence on long-term survival in patients with stages I–III gastric cancer in the Netherlands. Int J Cancer.

[4] Karimi P, Islami F, Anandasabapathy S, Freedman ND, Kamangar F (2014). Gastric cancer: descriptive epidemiology, risk factors, screening, and prevention. Cancer Epidemiol Biomark Prev.

[5] Van Cutsem E, Sagaert X, Topal B, Haustermans K, Prenen H (2016). Gastric cancer. Lancet.

[6] Esteller M (2011). Non-coding RNAs in human disease. Nat Rev Genet.

[7] Mercer TR, Dinger ME, Mattick JS (2009). Long non-coding RNAs: insights into functions. Nat Rev Genet.

[8] Huarte M (2015). The emerging role of lncRNAs in cancer. Nat Med.

[9] Sun W, Yang Y, Xu C, Xie Y, Guo J (2016). Roles of long noncoding RNAs in gastric cancer and their clinical applications. J Cancer Res Clin Oncol.

[10] Huang Y, Zhang J, Hou L, Wang G, Liu H, Zhang R (2017). LncRNA AK023391 promotes tumorigenesis and invasion of gastric cancer through activation of the PI3K/Akt signaling pathway. J Exp Clin Cancer Res.

[11] Fu M, Huang Z, Zang X, Pan L, Liang W, Chen J (2018). Long noncoding RNA LINC00978 promotes cancer growth and acts as a diagnostic biomarker in gastric cancer. Cell Prolif.

[12] YiRen H, YingCong Y, Sunwu Y, Keqin L, Xiaochun T, Senrui C (2017). Long noncoding RNA MALAT1 regulates autophagy associated chemoresistance via miR-23b-3p sequestration in gastric cancer. Mol Cancer.

[13] Wu H, Hu Y, Liu X, Song W, Gong P, Zhang K (2017). LncRNA TRERNA1 function as an enhancer of SNAI1 promotes gastric Cancer metastasis by regulating epithelial-Mesenchymal transition. Mol Ther Nucleic Acids.

[14] Fan Y, Shi Y, Lin Z, Huang X, Li J, Huang W, et al. miR-9-5p Suppresses Malignant Biological Behaviors of Human Gastric Cancer Cells by Negative Regulation of TNFAIP8L3. Dig Dis Sci. 2019. 10.1007/s10620-019-05626-2 31140050

[15] Hong KS, Kim H, Kim SH, Kim M, Yoo J (2019). Calponin 3 regulates cell invasion and doxorubicin resistance in gastric Cancer. Gastroenterol Res Pract.

[16] Xiong Y, Zhou L, Su Z, Song J, Sun Q, Liu SS (2019). Longdaysin inhibits Wnt/β-catenin signaling and exhibits antitumor activity against breast cancer. OncoTargets Ther..

[17] Zheng S, Wu H, Wang F, Lv J, Lu J, Fang Q (2019). The oncoprotein HBXIP facilitates metastasis of hepatocellular carcinoma cells by activation of MMP15 expression. Cancer Manag Res.

[18] Kang X, Kong F, Huang K, Li L, Li Z, Wang X (2019). LncRNA MIR210HG promotes proliferation and invasion of non-small cell lung cancer by upregulating methylation of CACNA2D2 promoter via binding to DNMT1. OncoTargets Ther.

[19] Liu B, Sun L, Liu Q, Gong C, Yao Y, Lv X (2015). A Cytoplasmic NF-κB Interacting Long Noncoding RNA Blocks IκB Phosphorylation and Suppresses Breast Cancer Metastasis. Cancer Cell.

[20] Dong J, Wang Q, Li L, Xiao-Jin Z (2018). Upregulation of long non-coding RNA small Nucleolar RNA host gene 12 contributes to cell growth and invasion in cervical Cancer by acting as a sponge for MiR-424-5p. Cell Physiol Biochem.

[21] Wang P, Ning S, Zhang Y, Li R, Ye J, Zhao Z (2015). Identification of lncRNA-associated competing triplets reveals global patterns and prognostic markers for cancer. Nucleic Acids Res.

[22] Shimura T, Takenaka Y, Tsutsumi S, Hogan V, Kikuchi A, Raz A (2004). Galectin-3, a novel binding partner of β-catenin. Cancer Res.

[23] Hu X-Y, Hou P-F, Li T-T, Quan H-Y, Li M-L, Lin T (2018). The roles of Wnt/β-catenin signaling pathway related lncRNAs in cancer. Int J Biol Sci.

[24] Zhu Q, Lu G, Luo Z, Gui F, Wu J, Zhang D (2018). CircRNA circ_0067934 promotes tumor growth and metastasis in hepatocellular carcinoma through regulation of miR-1324/FZD5/Wnt/β-catenin axis. Biochem Biophys Res Commun.

[25] Xian X, Tang L, Wu C, Huang L (2018). miR-23b-3p and miR-130a-5p affect cell growth, migration and invasion by targeting CB1R via the Wnt/β-catenin signaling pathway in gastric carcinoma. OncoTargets Ther..

[26] Takemaru K-I, Yamaguchi S, Lee YS, Zhang Y, Carthew RW, Moon RT (2003). Chibby, a nuclear β-catenin-associated antagonist of the Wnt/wingless pathway. Nat..

[27] Barker N, Hurlstone A, Musisi H, Miles A, Bienz M, Clevers H (2001). The chromatin remodelling factor Brg-1 interacts with beta-catenin to promote target gene activation. EMBO J.

[28] Bhan A, Soleimani M, Mandal SS (2017). Long noncoding RNA and Cancer: a new paradigm. Cancer Res.

[29] Schmitt AM, Chang HY (2016). Long noncoding RNAs in Cancer pathways. Cancer Cell.

[30] Gong P, Qiao F, Wu H, Cui H, Li Y, Zheng Y (2018). LncRNA UCA1 promotes tumor metastasis by inducing miR-203/ZEB2 axis in gastric cancer. Cell Death Dis.

[31] Zhou J, Shi J, Fu X, Mao B, Wang W, Li W (2018). Linc00441 interacts with DNMT1 to regulate RB1 gene methylation and expression in gastric cancer. Oncotarget..

[32] Zhuo W, Liu Y, Li S, Guo D, Sun Q, Jin J (2019). Long Noncoding RNA <em>GMAN</em>, Up-regulated in Gastric Cancer Tissues, Is Associated With Metastasis in Patients and Promotes Translation of Ephrin A1 by Competitively Binding <em>GMAN-AS</em>. Gastroenterol.

[33] Huang C, Hu Y-W, Zhao J-J, Ma X, Zhang Y, Guo F-X (2016). Long noncoding RNA HOXC-AS1 suppresses ox-LDL-induced cholesterol accumulation through promoting HOXC6 expression in THP-1 macrophages. DNA Cell Biol.

[34] Soucek L, Whitfield J, Martins CP, Finch AJ, Murphy DJ, Sodir NM (2008). Modelling Myc inhibition as a cancer therapy. Nat..

[35] Calcagno D-Q, Leal M-F, Assumpcao P-P, Smith M-A-C, Burbano R-R (2008). MYC and gastric adenocarcinoma carcinogenesis. World J Gastroenterol.

[36] Khanna A, Böckelman C, Hemmes A, Junttila MR, Wiksten JP, Lundin M (2009). MYC-dependent regulation and prognostic role of CIP2A in gastric cancer. J Natl Cancer Inst.

[37] Noh Ji Heon, Kim Kyoung Mi, McClusky Waverly G., Abdelmohsen Kotb, Gorospe Myriam (2018). Cytoplasmic functions of long noncoding RNAs. Wiley Interdisciplinary Reviews: RNA.

[38] Sanchez-Mejias A, Tay Y (2015). Competing endogenous RNA networks: tying the essential knots for cancer biology and therapeutics. J Hematol Oncol.

[39] Abdolvahabi Z, Nourbakhsh M, Hosseinkhani S, Hesari Z, Alipour M, Jafarzadeh M, et al. MicroRNA-590-3P suppresses cell survival and triggers breast cancer cell apoptosis via targeting sirtuin-1 and deacetylation of p53. J Cell Biochem. 2018. 10.1002/jcb.28211 30520099

[40] Gu L, Lu LS, Zhou DL, Liu ZC. UCA1 promotes cell proliferation and invasion of gastric cancer by targeting CREB1 sponging to miR-590-3p. Cancer Med. 2018. 10.1002/cam4.1310 PMC5911610 29516678

[41] Huo H, Tian J, Wang R, Li Y, Qu C, Wang N (2018). Long non-coding RNA NORAD upregulate SIP1 expression to promote cell proliferation and invasion in cervical cancer. Biomed Pharmacother.

[42] Zheng ZQ, Li ZX, Zhou GQ, Lin L, Zhang LL, Lv JW, et al. Long non-coding RNA FAM225A promotes nasopharyngeal carcinoma tumorigenesis and metastasis by acting as ceRNA to sponge miR-590-3p/miR-1275 and upregulate ITGB3. Cancer Res. 2019. 10.1158/0008-5472.CAN-19-0799 31331909

[43] Jeong O-S, Chae Y-C, Jung H, Park SC, Cho S-J, Kook H (2016). Long noncoding RNA linc00598 regulates CCND2 transcription and modulates the G1 checkpoint. Sci Rep.

[44] McHugh CA, Chen C-K, Chow A, Surka CF, Tran C, McDonel P (2015). The Xist lncRNA interacts directly with SHARP to silence transcription through HDAC3. Nat..

[45] Sun T-T, He J, Liang Q, Ren L-L, Yan T-T, Yu T-C (2016). LncRNA GClnc1 promotes gastric carcinogenesis and may act as a modular scaffold of WDR5 and KAT2A complexes to specify the histone modification pattern. Cancer Discov.

